# Optimisation of cognitive performance in rodent operant (touchscreen) testing: Evaluation and effects of reinforcer strength

**DOI:** 10.3758/s13420-017-0260-7

**Published:** 2017-02-15

**Authors:** Benjamin U. Phillips, Christopher J. Heath, Zofia Ossowska, Timothy J. Bussey, Lisa M. Saksida

**Affiliations:** 1 Department of Psychology and MRC/Wellcome Trust Behavioural and Clinical Neuroscience Institute, University of Cambridge, Downing Street, Cambridge, CB2 3EB UK; 2 School of Life, Health and Chemical Sciences, The Open University, Walton Hall Milton Keynes, MK7 6AA UK; 3 Molecular Medicine Research Laboratories, Robarts Research Institute & Department of Physiology and Pharmacology, Schulich School of Medicine & Dentistry, Western University, London, ON Canada; 4 The Brain and Mind Institute, Western University, London, ON Canada

**Keywords:** Touchscreen, Mouse, Progressive ratio, Fixed ratio, Rate analysis, Reinforcer

## Abstract

Operant testing is a widely used and highly effective method of studying cognition in rodents. Performance on such tasks is sensitive to reinforcer strength. It is therefore advantageous to select effective reinforcers to minimize training times and maximize experimental throughput. To quantitatively investigate the control of behavior by different reinforcers, performance of mice was tested with either strawberry milkshake or a known powerful reinforcer, super saccharin (1.5% or 2% (w/v) saccharin/1.5% (w/v) glucose/water mixture). Mice were tested on fixed (FR)- and progressive-ratio (PR) schedules in the touchscreen-operant testing system. Under an FR schedule, both the rate of responding and number of trials completed were higher in animals responding for strawberry milkshake versus super saccharin. Under a PR schedule, mice were willing to emit similar numbers of responses for strawberry milkshake and super saccharin; however, analysis of the *rate* of responding revealed a significantly higher rate of responding by animals reinforced with milkshake versus super saccharin. To determine the impact of reinforcer strength on cognitive performance, strawberry milkshake and super saccharin-reinforced animals were compared on a touchscreen visual discrimination task. Animals reinforced by strawberry milkshake were significantly faster to acquire the discrimination than animals reinforced by super saccharin. Taken together, these results suggest that strawberry milkshake is superior to super saccharin for operant behavioral testing and further confirms that the application of response rate analysis to multiple ratio tasks is a highly sensitive method for the detection of behavioral differences relevant to learning and motivation.

## Introduction

Behavioral neuroscience frequently employs rodent models to determine the effects of particular manipulations on behavior and cognition. This often involves assessment in tasks based on operant conditioning (Keesey & Goldstein, 1968; Markou et al., 2013), including those available in the rodent touchscreen apparatus (Horner et al., 2013; Hvoslef-Eide et al., 2015; Mar et al., 2013; Oomen et al., 2013). The performance of rodents on such tasks can be affected by the properties and quantity of reinforcer (Adams & Dickinson, 1981; Eagle, Humby, Dunnett, & Robbins, 1999; Skjoldager, Pierre, & Mittleman, 1993) across a wide range of tasks and manipulations (G. S. Brown & Geoffrey, 2009; Chudasama & Robbins, 2006; Hutsell & Newland, 2013). Thus, the choice of reinforcer can be important in such studies, for a number of reasons. For example, researchers may wish to select reinforcers that elicit high rates of responding in order to minimize training times, thereby enhancing throughput. Therefore, the efficiency of a particular operant testing method can be increased by assessing the impact of different reinforcer options on task performance.

The operant touchscreen testing platform allows a wide range of cognitive abilities (e.g., working memory, attention, associative learning, and cognitive flexibility) to be assessed in the same physical apparatus, using the same types of stimuli and responses (Bussey et al., 2008; Horner et al., 2013; Leising, Wolf, & Ruprecht, 2013; Mar et al., 2013; Oomen et al., 2013; Pineño, 2014). This method is gaining in popularity and has emerged as a widely adopted approach for the study of rodent behavior. The standard reinforcer used in touchscreen tasks is strawberry milkshake. Informal observation suggests strawberry milkshake is a strikingly powerful reinforcer in a wide variety of tasks for the assessment of cognition and behavior (Horner et al., 2013; Mar et al., 2013; Oomen et al., 2013). However, no controlled study has tested whether animals will work harder for strawberry milkshake, or will perform better in cognitive tasks with this reinforcer compared to other available liquid reinforcers. Thus, the present study formally assessed the reinforcement strength of strawberry milkshake by comparing it against a powerful liquid reinforcer: a saccharin and glucose mixture (super saccharin) (Blasio et al., 2012; Sabino et al., 2011; Valenstein, Cox, & Kakolewski, 1967).

Motivated behavior in rodents is often investigated via the use of ratio schedules, which typically require emission of a defined number of responses for a fixed quantity of reinforcer. Fixed ratio (FR) schedules require an invariant number of responses per reinforcer whereas in progressive ratio (PR) schedules the response requirement increments with each reinforcer earned. PR schedules, as originally conceived, measure reward strength, but have since become the canonical test of motivation in behavioral neuroscience (Hodos, 1961; Markou et al., 2013). Ratio schedule performance has previously been shown to be sensitive to manipulation of reward magnitude, palatability, and reinforcer state (Eagle et al., 1999; Hodos, 1961; Hutsell & Newland, 2013). It has also been demonstrated that performance is sensitive to endogenous manipulations, including degree of food restriction and various pharmacologic interventions (Aberman & Salamone, 1999; Aberman, Ward, & Salamone, 1998; Eagle et al., 1999).

To objectively compare the strawberry milkshake and super saccharin reinforcers, we used the recently validated touchscreen FR and PR schedules for the mouse (Heath, Bussey, & Saksida, 2015; Heath, Phillips, Bussey, & Saksida, 2016). Responding for the reinforcers diverged substantially depending on the schedule, with large differences between strawberry milkshake and both concentrations of super saccharin apparent on the FR schedule. Animals responding for strawberry milkshake under the PR schedule did not significantly differ from 1.5% super saccharin as measured by breakpoint, but analysis of *rate* of responding revealed clear differences, milkshake supporting significantly higher rates of responding than super saccharin.

To determine if the observed difference in reinforcer strength had any impact on the performance of a touchscreen cognitive assessment, a comparison was performed using visual pairwise discrimination learning (Horner et al., 2013). Animals reinforced by strawberry milkshake acquired the task significantly more quickly and committed significantly fewer errors than super saccharin-reinforced animals. These results demonstrate the strength of strawberry milkshake as a reinforcer in operant tasks, reaffirm a substantive link between cognitive processes and motivation (Avlar et al., 2015), and support the employment of within-session analysis of ratio task performance to detect subtle differences in behavior (Bradshaw & Killeen, 2012).

## Materials and methods

### Animals

Male C57BL/6 mice (n = 30; Charles River Laboratories, Margate, UK) housed in groups of four (one group of two) between 8–10 weeks of age were habituated to the housing room (12-h light/dark cycle, lights off 0700) in the animal facility for 7 days after arrival. All animals were then weighed for three consecutive days to establish mean free feeding weights. Mild food restriction was initiated and sustained at 85–90% of free feeding weight by daily provision of specific amounts of standard laboratory chow (RM3, Special Diet Services). Drinking water was available *ad libitum* throughout the study. All animals were tested once daily 5–7 days a week during the dark phase. One animal was culled on welfare grounds following FR testing and is consequently not present in subsequent behavioral procedures or analyses. All procedures were performed in accordance with the United Kingdom Animals (Scientific Procedures) Act (1986) and the United Kingdom Animals (Scientific Procedures) Act (1986) Amendment Regulations 2012.

### Apparatus

All training and testing was carried out in standard Bussey-Saksida mouse touchscreen chambers (Campden Instruments Ltd, Loughborough, Leicestershire, UK). These chambers have been described in detail elsewhere (Horner et al., 2013; Mar et al., 2013; Oomen et al., 2013). Briefly, the trapezoidal touchscreen-operant chamber is housed inside a sound-attenuating chamber. Responses at the touchscreen (12.1 in.; resolution 800 x 600) are made by breaking IR beams positioned close to the surface of the screen. A black perspex mask was placed in front of the touchscreen in order to protect the edges of the screen and focus responding to the appropriate spatial location. In this study, the standard ‘5-choice’ mask (Campden Instruments Ltd) was used in both the PR and the FR assessments, whilst a standard two-hole mask was used for the pairwise discrimination task (Heath et al., 2015, 2016; Horner et al., 2013). All behavioral programs were controlled and implemented by ABET II Touch software (Campden Instruments Ltd) and Whisker Server (Cardinal & Aitken, 2010).

### Ratio task training and procedures

Two types of reinforcer were used in this study: strawberry milkshake (Yazoo Strawberry UHT milkshake; FrieslandCampinaUK, Horsham, UK) and two concentrations of super saccharin (Blasio et al., 2012): 1.5% or 2% (w/v) saccharin with a fixed 1.5% (w/v) glucose in tap water (Sigma-Aldrich, Dorset, UK). All animals were pseudo-randomly designated a liquid reinforcer so all groups were equally sized. These concentrations of super saccharin were selected based on our pilot studies and previous literature reporting that similar concentrations of super saccharin are capable of sustaining operant behavior in rodents (Blasio et al., 2012; Sabino et al., 2011; Valenstein et al., 1967).

The training procedure for touchscreen FR and PR has been described in detail elsewhere (Heath et al., 2015, 2016). Briefly, all animals were initially habituated to the chambers for 20-min sessions over two consecutive days. Two hundred microliters of the allocated reinforcer was provided in the reward collection magazine. The criterion for habituation was consumption of the reinforcer in at least one of these sessions. Following habituation mice were trained to emit responses at the screen for one session. During these sessions, the central response location was illuminated with a white square for 30 s. Following this, the square was removed and the reward feeder pump switched on for 800 ms to deliver 20 μl of the designated reinforcer to the reward collection magazine. If the illuminated square was touched triple the reinforcer volume was delivered to the magazine. All animals were required to consume 30 reinforcers in 60 min. Upon completion of this stage, animals were moved on to an FR1 schedule which required the completion of 30 trials in 60 min. Animals were then transferred to an FR2 schedule which required completion of 15 trials in 60 min and then an FR3 schedule which required completion of ten trials in 60 min. All schedules required emission of 30 touchscreen responses in total. All animals were subsequently moved to an FR5 schedule which required completion of 30 trials in 60 min for two consecutive sessions. Once this stage had been completed, animals were introduced to a PR4 schedule in which the response requirement was increased on each trial according to a linear ramp (1, 5, 9, 13, 17, 21, etc.). These sessions were terminated after 60 min or 5 min of inactivity. Following PR4 assessment, all animals were tested on an FR5 schedule for a single session. This session was terminated after 60 min had elapsed and animals were permitted to complete as many trials as possible.

### Pairwise discrimination training and procedures

Touchscreen pairwise discrimination was conducted as described previously (Horner et al., 2013). Briefly, animals were trained to initiate stimulus presentation by entering the reward magazine when illuminated and that an incorrect screen touch would be punished with a 5-s timeout and house light illumination. For discrimination acquisition, animals were required to select between two concurrently presented visual stimuli. These were diagonal bands of black and white stripes angled either left or right. One stimulus was allocated as the S+ (always reinforced with 20 μl of the designated reinforcer) and the other as the S- (always punished with a 5-s timeout and house light illumination). The S+ was counterbalanced between subjects within each reinforcer group. Additionally, the S+ and S- presentation location was pseudorandomly selected between trials such that each stimulus would be presented equally in each response location across each session. All animals were tested once daily until they had reached criterion (defined as two consecutive sessions with ≥ 80% correct). Each session terminated either after 30 trials had been completed or after 1 h had elapsed.

### Data analysis

For the PR assessment breakpoint (the number of responses emitted in the last trial the animal successfully completed) and the total number of screen touches emitted in the session were recorded. Additionally, values for total response time (time from the first screen touch to the last screen touch of a discrete trial), post-reinforcement pause (time from reward collection to the first screen touch of the next trial) and inter-reinforcer interval (time from the first touch of a trial to the first touch of the next trial) per trial were collected. For the FR schedule, the total number of trials completed was collected instead of breakpoint. Two versions of response rate were calculated for between-group analysis and visualization for both ratio schedules. This required conversion of total response times and inter-reinforcer interval to rate per trial (Bradshaw & Killeen, 2012; Olarte-Sánchez, Valencia-Torres, Cassaday, Bradshaw, & Szabadi, 2015).

PR data were analyzed based on mean performance across two consecutive sessions. Data were analyzed using one-way ANOVA with a significance level of *p* < 0.05 unless otherwise indicated. Post-hoc tests were carried out using Tukey’s HSD test. All data were tested for homogeneity of variance using Levene’s test. Rate measures for each animal were fitted with a negative exponential function y = a^(-b*x)) for PR where y represents response rate, x represents trials, -b represents decay and a represents the y intercept as previously reported (Bailey et al., 2015; Ward, Simpson, Kandel, & Balsam, 2011). The values of –b and a were extracted for each animal for both total response time and inter-reinforcer interval rate. FR response rate measures were fitted with the parabolic function y = b*(x)^2 + a. Values for the intercept (a) and decay (b) parameter of each function were then extracted and tested for between-group statistical significance.

Pairwise discrimination percentage correct data was fitted with a linear-mixed model using the package ‘lme4’ for the R software package for statistical computing as this class of model tolerates subjects reaching criterion at different rates through repeated measures time points (Boisgontier & Cheval, 2016). Predictor variables of session number and reinforcer were treated as fixed effects whilst session and subject (individual animal) were treated as crossed random effects. The fitted model was then subjected to the ANOVA function from the ‘lmerTest’ package for R to obtain Sattherwaite estimated F and *p*-values for main effects and interactions. Additionally, the fitted model was compared with a null model (intercept only) that did not include fixed effects as predictors with ANOVA to evaluate goodness of fit. Post-hoc comparison on the full model was carried out using the ‘lsmeans’ package in R.

All statistical analyses were performed using the R software package for statistical computing (www.r-project.org). All data are presented as mean ± standard error of the mean. Asterisks indicate statistical significance in all figure legends.

## Results

PR breakpoint analysis indicated no differences between strawberry milkshake and 1.5% super saccharin. However, strawberry milkshake supports significantly higher PR performance than 2% super saccharin.

**Fig. 1:**
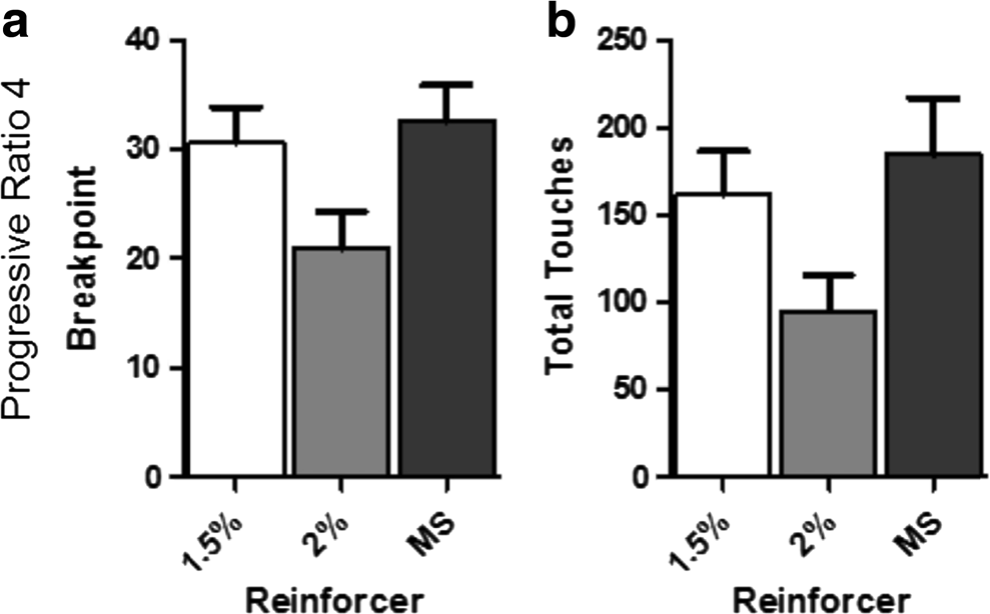
Reinforcer type affects performance PR. (**A**) Mean PR4 breakpoint under different reinforcers. (**B**) Mean total touches emitted on a PR4 schedule under different reinforcers

Reinforcer type significantly modulated PR4 performance as measured by breakpoint (F(2,27) = 3.61, *p* < 0.05), with analysis of total touches indicating a similar trend (F(2,27) = 3.05, *p* < 0.06) (Fig. 1(a) and (b)). Post-hoc comparison revealed that strawberry milkshake supported a higher breakpoint than 2% super saccharin (*p* < 0.05). The PR performance of animals reinforced by strawberry milkshake and 1.5% super saccharin did not differ significantly as measured by breakpoint. Similarly, the breakpoints supported by 1.5% and 2% super saccharin did not significantly differ.

PR response rate analysis reveals underlying differences in the pattern of responding for strawberry milkshake and super saccharin.

**Fig. 2:**
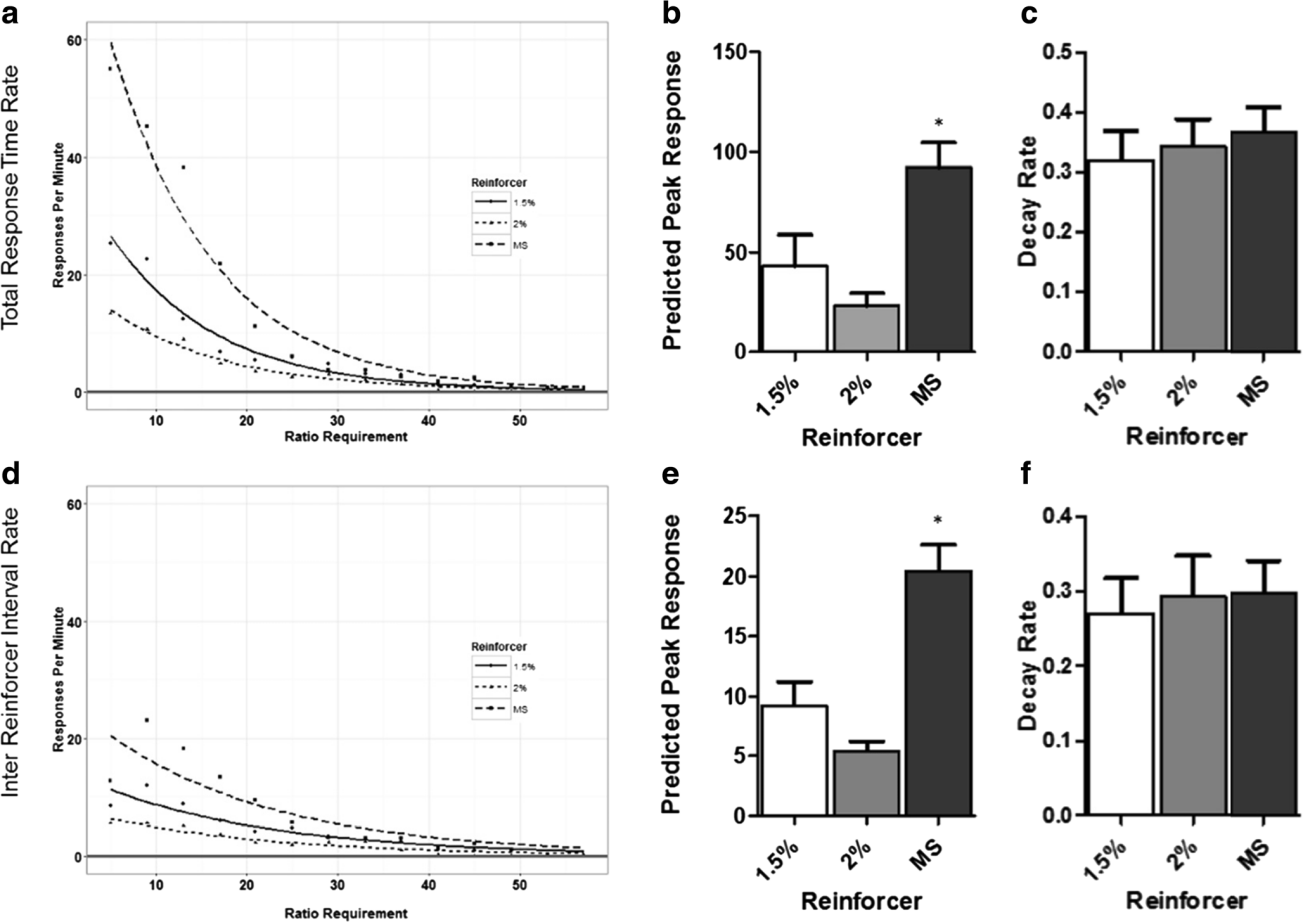
Reinforcer type affects within-session response measures on PR. (**A**) PR group mean total response time rate of responding from second trial onwards. Data are fitted with the negative exponential y = a^exp(-b*x). (**B**) Mean fitted predicted peak total response time response rate. (**C**) Mean fitted total response time rate of decay. (**D**) PR group mean inter-reinforcer interval rate of responding from second trial onwards. Data are fitted with the negative exponential y = a^exp(-b*x). (**E**) Mean fitted predicted peak inter-reinforcer interval response rate. (**F**) Mean fitted inter-reinforcer interval rate of decay

Analysis of total response time (Fig. 2(a–c)) and inter-reinforcer interval rates (Fig. 2(d–f)) revealed underlying differences in PR performance not fully captured by the prior breakpoint analysis.

In order to understand the response trajectories associated with each reinforcer, individual sessions were fitted with the negative exponential function y = a^exp(-b*x). A significant effect of reinforcer type was detected on total response time peak response rate predicted by the fitted equation y = a^(-b*n)) (F(2,26) = 9.94, *p* < 0.001) (Fig. 2(b)). Post-hoc analysis of this measure revealed that strawberry milkshake supported a significantly higher predicted peak response rate than both 1.5% (*p* < 0.005) and 2% (*p* < 0.005) super saccharin. No significant difference was detected between 1.5% and 2% super saccharin. No significant effect of reinforcer type was detected on response rate decay (Fig. 2(c)).

A significant effect of reinforcer type was detected on predicted peak response inter-reinforcer interval rate (F(2,25) = 6.83, *p* < 0.001) (Fig. 2(e)). Post-hoc testing revealed that strawberry milkshake supported a higher predicted peak response than 2% super saccharin (*p* < 0.005) and trended toward supporting a higher predicted peak response than 1.5% super saccharin (*p* < 0.08). No significant effect of reinforcer type was detected on response rate decay (Fig. 2(f)).

Strawberry milkshake supports a higher response rate and overall operant output than super saccharin on an unrestricted FR schedule.

**Fig. 3:**
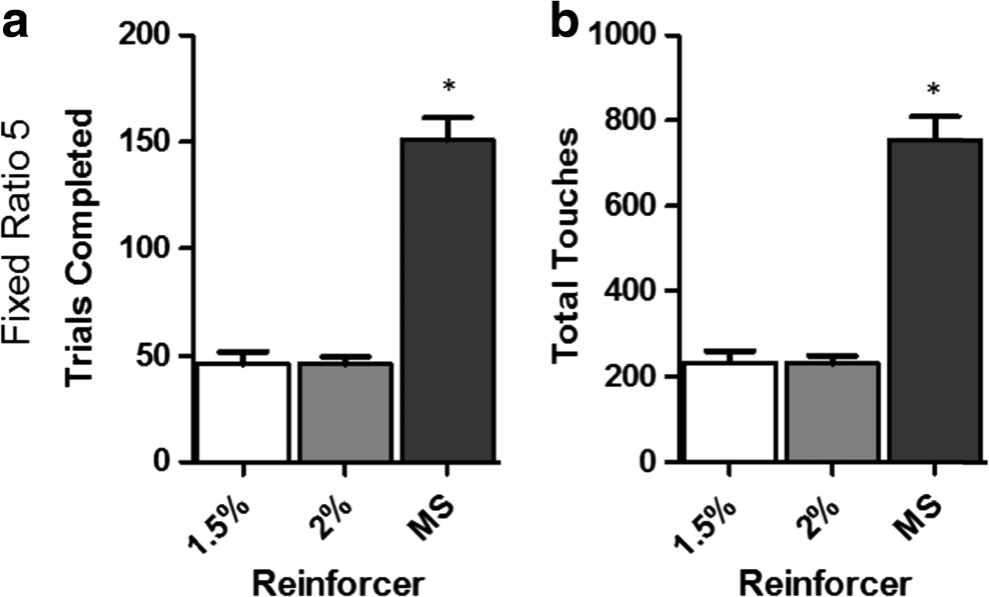
Reinforcer type affects performance on FR. (**A**) Mean total trials completed on an FR5 schedule under different reinforcers. (**B**) Mean total touches emitted on an FR5 schedule under different reinforcers

To further characterize the effect of qualitatively different reinforcers on operant behavior, we tested all mice on an FR5 schedule with no trial limit. A main effect of reinforcer type was detected on total trials completed (F(2,26) = 67.39, *p* < 0.001) and total touches emitted (F(2,26) = 66.5, *p* < 0.001) (Fig. 3(a) and (b)). Post-hoc analysis revealed that mice reinforced by strawberry milkshake completed significantly more trials than those reinforced with either 1.5% (*p* < 0.001) or 2% (*p* < 0.001) super saccharin. There was no difference in trial completion between the 1.5% and 2% groups. Post-hoc analysis of total touches indicated that animals reinforced by strawberry milkshake emitted significantly more touches than 1.5% (*p* < 0.001) and 2% (*p* < 0.001) super saccharin. No significant differences were observed between 1.5% and 2% super saccharin on this measure.

**Fig. 4:**
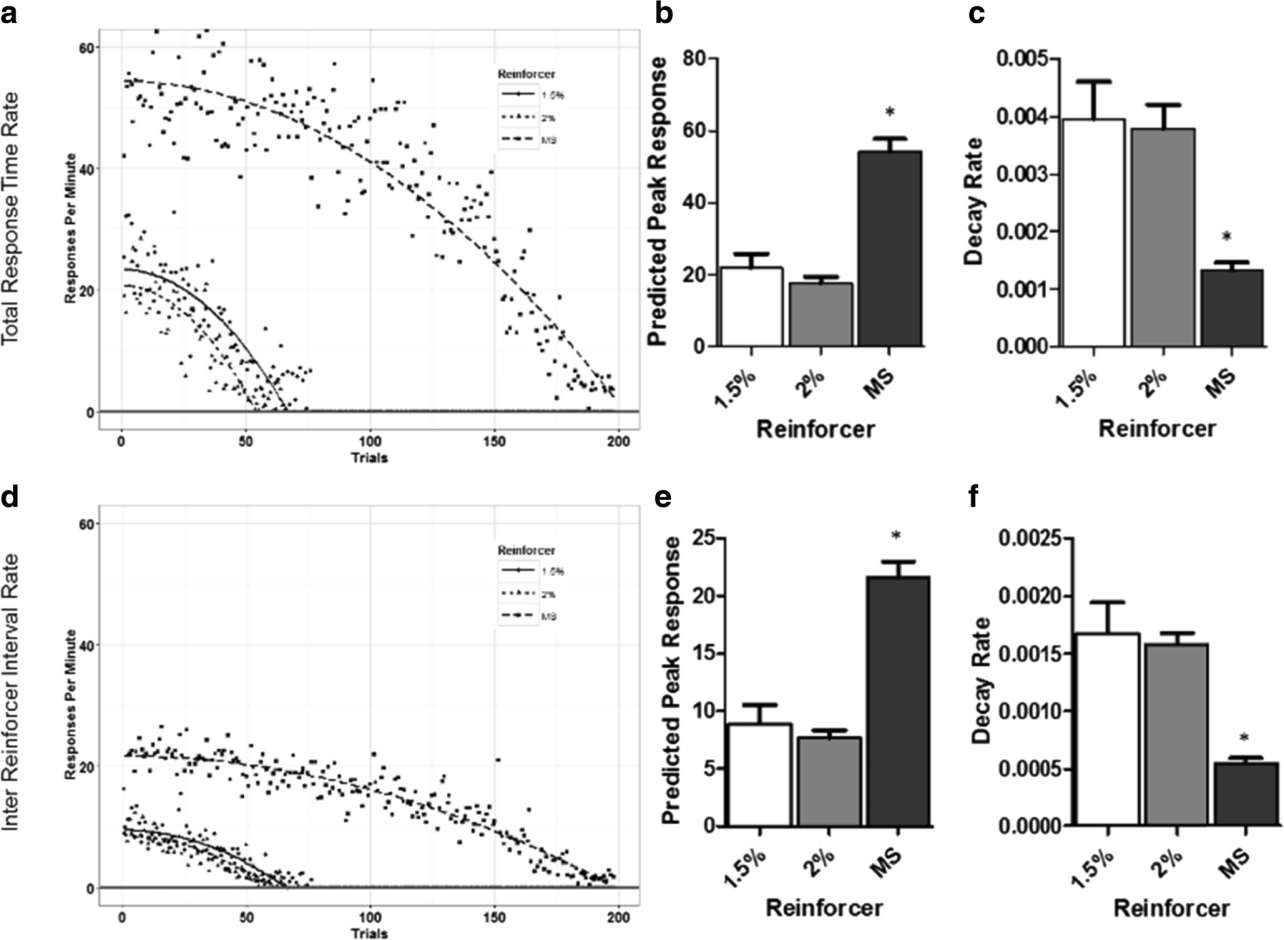
Reinforcer type affects within-session response measures on FR. (**A**) FR5 group mean total response time rate of responding. Data are fitted with the function y = -b*(x)^2 + a. (**B**) Mean fitted predicted peak total response time response rate. (**C**) Mean fitted total response time rate of decay. (**D**) FR5 group mean inter-reinforcer interval rate of responding. Data are fitted with the function y = -b*(x)^2 + a. (**E**) Mean fitted predicted peak inter-reinforcer interval response rate. (**F**) Mean fitted inter-reinforcer interval rate of decay

Analysis of the underlying response pattern revealed differences in both total response time rates and inter-reinforcer interval rates on FR. Response rates for both inter-reinforcer interval and total response time per animal were fitted with the parabolic function y = -b*(x)^2 + a (Fig. 4(a–d)). The values for a and –b were extracted for each animal and analyzed for between-group differences. A significant effect of reinforcer type on both the predicted peak (F(2,26) = 39.47, *p* < 0.001) and decay (F(2,26) = 10.97, *p* < 0.001) coefficients of the total response time rate was found (Fig. 4(b) and (c)). Post hoc tests revealed that strawberry milkshake supported a higher predicted peak response rate than both 1.5% (*p* < 0.001) and 2% (*p* < 0.001) super saccharin (Fig. 4(b)). Additionally, responding reinforced by strawberry milkshake decayed at a significantly slower rate than both 1.5% (*p* < 0.001) and 2% (*p* < 0.005) super saccharin (Fig. 4(c)).

Similarly, a significant effect of reinforcer type on both predicted peak responding (F(2,26) = 34.66, *p* < 0.001) and decay (F(2,26) = 13.36, *p* < 0.001) of the inter-reinforcer interval rate was detected (Fig. 4(e) and (f)). Strawberry milkshake supported a higher predicted peak response than both 1.5% (*p* < 0.001) and 2% (*p* < 0.001) super saccharin (Fig. 4(e)); 1.5% and 2% super saccharin did not differ significantly on this measure. Similarly, responding for strawberry milkshake decayed at a slower rate than both 1.5% (*p* < 0.001) and 2% (*p* < 0.001) super saccharin (Fig. 4(f)); 1.5% and 2% super saccharin did not significantly differ on this measure.

Strawberry milkshake supports a significantly faster rate of acquisition than super saccharin on a touchscreen visual discrimination.

**Fig. 5:**
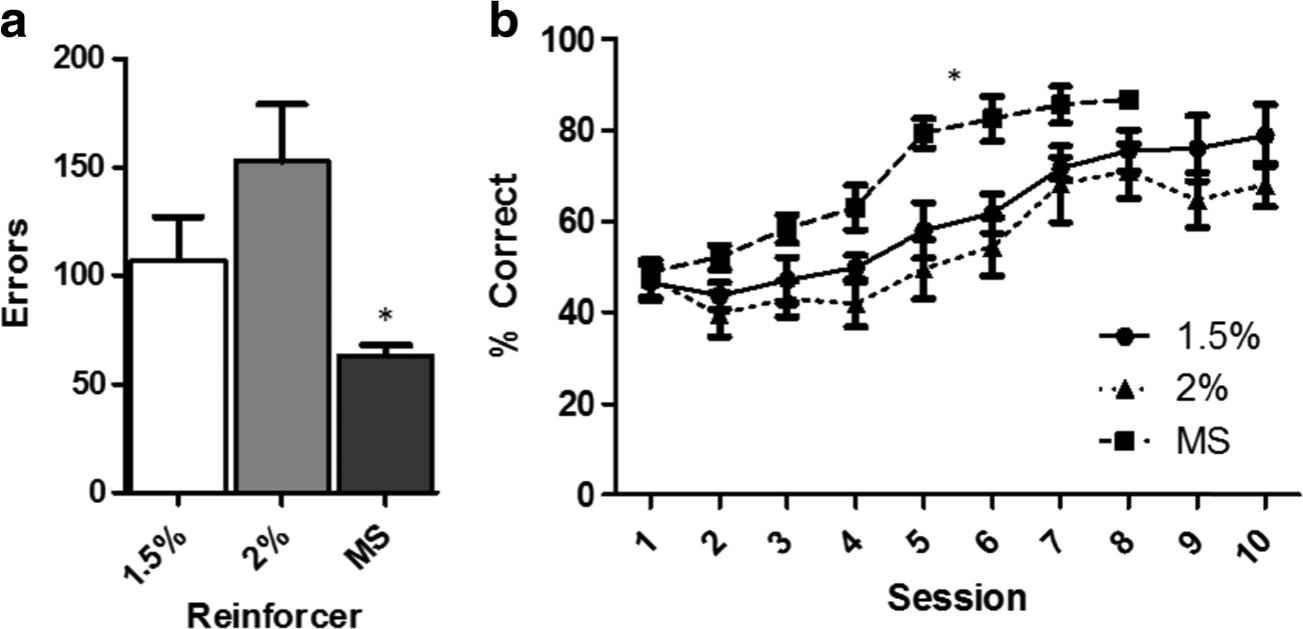
Reinforcer type affects performance on a visual discrimination task. (**A**) Number of errors made before criterion reached. (**B**) Percent correct by session per reinforcer for the first ten sessions

To determine the extent to which differences in reinforcer properties affect learning, we tested all mice on a touchscreen pairwise discrimination task. Reinforcer significantly affected the number of errors made to criterion (F(2, 26) = 5.69, *p* < 0.001) (Fig. 5(a)). Post-hoc comparison between groups revealed that animals reinforced by strawberry milkshake made significantly fewer errors than animals reinforced by 2% super saccharin (*p* < 0.05) but not 1.5% super saccharin (*p* < 0.25).

A linear mixed model was fitted to the first ten sessions of the discrimination acquisition with reinforcer and session designated as fixed effects and session nested within subject (individual animal) designated as random effects. A significant effect of session was detected (F(1,13.16) = 94.67, *p* < 0.0001) (Fig. 5(b)). In addition, a significant interaction between reinforcer and session was detected (F(2, 193.74) = 8.61, *p* < 0.001). No significant effect of reinforcer (F(2,94.83) = 0.11, *p* = 0.9) was detected. Post-hoc testing detected a significant difference between the strawberry milkshake group and both the 1.5% (*p* < 0.001) and 2% (*p* < 0.001) groups. No significant difference was detected between the two saccharin groups (*p* = 0.25). In addition, the full model was a significantly better fit than the null model (intercept only) (*p* < 0.0001).

## Discussion

Cognition and behavior are frequently studied in mice using operant tasks, in which responses can be reinforced by a pleasurable outcome. It has been reliably shown that behavior in such tasks can be modulated via manipulation of reinforcer characteristics (Baron, Mikorski, & Schlund, 1992; Hutsell & Newland, 2013; Olarte-Sánchez et al., 2015; Skjoldager et al., 1993). There is, therefore, a clear requirement for greater understanding of the influence of reinforcers on operant behavior, both in order to select a suitable reinforcer for cognitive tests, and to better understand the mechanisms that govern behavior-reinforcer interactions. Similarly, it is instructive to examine these effects in the context of the rodent touchscreen apparatus as it becomes increasingly more widespread in behavioral neuroscience research laboratories. Therefore, we investigated the reinforcing properties of different reinforcers in the touchscreen FR and PR schedules (Heath et al., 2015, 2016) in C57BL/6 mice. We compared strawberry milkshake, which is typically used as the reinforcer in mouse touchscreen chambers in our laboratory (Horner et al., 2013; Mar et al., 2013; Oomen et al., 2013), and two concentrations of super saccharin, which is a known powerful reinforcer for supporting operant behavior in rodents (Blasio et al., 2012; Sabino et al., 2011; Valenstein et al., 1967).

Selective behavioral effects that were dependent upon reinforcer type and schedule of reinforcement were identified. Specifically, strawberry milkshake and 1.5% super saccharin did not significantly differ in total touches emitted or breakpoint on the PR schedule, suggesting equivalent reinforcer value (Hodos, 1961). Both of these reinforcers supported higher levels of performance than 2% super saccharin, potentially consistent with gustatory aversion to increasing concentrations of saccharin in rodents (Siviy & Reid, 1983). In contrast, on an FR5 schedule with unlimited trial availability, strawberry milkshake reinforcement led to animals completing significantly more trials and emitting more total touches than either concentration of super saccharin, which did not differ from one another. Following FR and PR, animals were tested on a touchscreen pairwise discrimination task. It was found that animals reinforced by strawberry milkshake learned significantly faster and made significantly fewer errors than the saccharin groups on this task. Our findings therefore indicate that reinforcers like strawberry milkshake can promote performance on cognitive tests, and the use of such reinforcers can serve to minimize training times and maximize experimental throughput in operant procedures. These conclusions are relevant to researchers using any type of reinforced behavior, but particularly to operant methods using liquid rewards and especially to those using touchscreens for which strawberry milkshake is used as the standard reinforcer.

### Detailed analysis of performance under different reinforcers

Measures derived from whole-session PR performance (e.g., breakpoint) did not discriminate the reinforcing strength of strawberry milkshake and 1.5% super saccharin. In contrast, strawberry milkshake was shown to be significantly more potent than both concentrations of super saccharin on the FR schedule which, unlike PR, is characterized by an invariant response requirement and relatively more frequent and predictable reinforcement delivery. This suggests that reinforcer efficacy is sensitive to the temporal coupling between response and outcome inherent in the behavioral schedule utilized. Since low-response requirement FR schedules are more closely associated with reliable and frequent presentation of reinforce, they are arguably better suited for understanding the influence of satiety and reinforcer feedback on the performance of cognitive tests, in which typically each correct response is reinforced. As reinforcement becomes increasingly infrequent under PR schedules, this increasingly limits the influence of reinforcer consumption on subsequent behavior and requires implementation of a cost-benefit analysis for a single reinforcing outcome which changes for each initiated trial. As such, the PR schedule measures the maximum effort expenditure for a single reinforcing outcome. Interpreted within this framework, the cost-benefit calculation for strawberry milkshake and 1.5% super saccharin did not differ under PR schedules, but differences between the reinforcers affected subsequent behavior owing to the high temporal density of reinforcement under FR schedules.

The importance of inter-reinforcement interval on ratio schedules is demonstrated by the finding that PR performance is sensitive to ratio step size (Covarrubias & Aparicio, 2008). This has highlighted potential limitations in measures derived from cumulative performance over entire ratio sessions, including breakpoint, and the suggestion that analysis of the temporal distribution of responding may be more informative (Bradshaw & Killeen, 2012). Consistent with this view, our analysis of the temporal distribution of responding revealed underlying differences in behavior on the touchscreen ratio schedules, depending on reinforcer.

Two versions of response rate across trials for both the touchscreen PR and FR schedules were calculated in this study. First, total response time rate captures the response rate as calculated from the first to last touch of a discrete trial. Second, inter-reinforcer interval rate captures the response rate as calculated from the first touch of a given trial to the first touch of the subsequent trial. It has been suggested that a dichotomy between these measures may capture differences in behavior related to motoric, mnemonic, and motivational processes (Bradshaw & Killeen, 2012).

Though there was no difference in PR breakpoint between strawberry milkshake and 1.5% super saccharin, we observed differences in these rate measures. Distinguishing between reinforcer efficacy and value can provide a theoretical framework for the interpretation of differences in rate (Hutsell & Newland, 2013; Rowlett, 2000). Reinforcer efficacy refers to the maximum response rate maintained by a reinforcer, which is a more relevant measure when comparing reinforcers for use in other operant tasks, whilst reinforcer value refers specifically to the maximum amount of effort exerted for a single reinforcer delivery (Hutsell & Newland, 2013; Rowlett, 2000). Strawberry milkshake consistently supported a higher predicted peak response rate than both concentrations of super saccharin, indicating that it acts with a higher degree of reinforcer efficacy. However, the maximum exertion emitted for a single reinforcer on the PR schedule did not differ between milkshake and 1.5% super saccharin, indicating that their value did not differ. Additionally, responding supported by strawberry milkshake decayed at a significantly slower rate on the FR but not the PR schedule. Since FR schedules are characterized by particularly dense and frequent reinforcement compared to PR schedules, this slower decay rate is indicative of reinforcer properties that maintain responding by acting through a consumption-response positive feedback loop. Theories of behavioral momentum may also help explain differences in behavioral parameters under fixed and progressive ratio schedules (J. A. Nevin & Grace, 2000; John A. Nevin, 2002). Within this framework, response rate can be equated to the momentum of a moving body, and reductions in rate comparable to the effects of disruptors on ongoing motion. Since PR schedules introduce disrupters in the form of increased response requirements for each subsequent reinforcer, they are subject to much higher rates of response rate decay, with the potential for corresponding dissociable effects on different schedules of reinforcement.

Moreover, temporal distribution of response analysis can complement traditional analysis of ratio tasks in a number of ways. Firstly, as was observed in this study, it may capture differences that are not apparent in commonly used measures such as breakpoint. Secondly, underlying differences in patterns of responding may be of relevance to motivational dysfunction in disease states. Peak rate of responding may reflect maximum energy output or motoric capacity. This is of particular relevance to movement disorders, including Parkinson disease and Huntington disease, in which patients and rodent models can develop motoric impairments (Abbs, Hartman, & Vishwanat, 1987; Baik et al., 1995; Carter et al., 1999; Taylor & Hansotia, 1983). Alternatively, it can capture differences in baseline or trait motivation, or the extent to which reinforcers act efficaciously. Motivational assessments in the clinic are typically carried out via questionnaires or surveys such as the neuropsychiatric inventory (Cummings et al., 1994). As such, this is a research area that has not received sufficient attention in humans. However, abnormal temporal patterns of responding have been observed in automated tasks in clinical populations including depression and schizophrenia (Arrondo et al., 2015; Murray et al., 2008). The recently developed EMOTICOM battery for the automated assessment of emotion, motivation, impulsivity, and social cognition holds promise for further quantitative investigation of this topic (Bland et al., 2016).

Response rate decay is reflective of the way in which behavior is controlled by reinforcer presentation. As related to disease state, this measure is of particular relevance to anhedonia, a symptom closely associated with loss of pleasure or interest in positive outcomes observed in conditions including depression, schizophrenia, and dementia (Barch, Treadway, & Schoen, 2014; G. S. Brown & Geoffrey, 2009; S. L. Brown, Schwartz, & Sweeney, 1978; Harrow, Grinker, Holzman, & Kayton, 1977; Kayton & Koh, 1975; Reichman & Coyne, 1995; Watson, Klett, & Lorei, 1970). Therefore, a reduction of pleasurable reinforcer influence on subsequent behavior may result in a faster rate of decay, as primary reinforcement characteristics are necessary to maintain responding under highly coupled schedules. Peak response rate and response rate decay are therefore suitable measures for identification of apathetic- and anhedonia-like phenotypes in rodent models. On PR schedules characterized by progressively longer bouts of responding between reinforcement, differences in decay rate may also reflect different levels of sensitivity to instrumental extinction processes (Ward et al., 2011). Such analysis has therefore also been suggested as a tool for isolating learning-related changes that may confound whole-session measures on PR schedules (Ward et al., 2011).

There are numerous mechanisms through which the effects of strawberry milkshake on motivated behavior and learning may be mediated. One explanation is that the palatability of strawberry milkshake may have resulted in enhanced behavioral activation, resulting in an increase in vigor under FR and PR and enhanced attention to task contingencies in the pairwise discrimination task. Alternatively, the nutritional characteristics of strawberry milkshake may have elicited substantial post-oral conditioning (Karen Ackroff, Dym, Yiin, & Sclafani, 2009; K. Ackroff & Sclafani, 2001), resulting in anticipatory behavioral activation. This proposal would explain the increased response rate observed at the beginning of ratio sessions. In addition, there is extensive evidence in support of the hypothesis that the nutritional characteristics of reinforcers are necessary for the maintenance of vigorous operant behavior (Beeler et al., 2012; McCutcheon, 2015). Thus, the nutritional aspects of strawberry milkshake, as compared to super saccharin, may have resulted in a higher level of engagement of reinforcement systems. The results of this study do not allow for selection between these competing explanations; future studies may seek to isolate the exact properties of strawberry milkshake that account for the observed reinforcement profile.

Classical learning theory provides a framework for understanding the influence of reinforcer on learning observed on the pairwise discrimination learning task. The Rescorla-Wagner model, Δv_x_ = αβ(λ-v_ax_), where Δv_x_ is the change in associative strength on a single trial, β is the association parameter, α is conditioned stimulus salience, λ is asymptotic conditioning and v_ax_ is the current associative strength of all conditioned stimuli, accounts for learning in terms of discrepancy between predicted and actual outcomes on a trial-by-trial basis (Rescorla & Wagner, 1972). The learning rate is partially determined by α, which captures the salience of the conditioned stimulus. In the present study, α may represent the coupling of reinforcer strength with the visual conditioned stimulus, providing a behavioral mechanism by which strawberry milkshake, the more salient reinforcer, supported a higher learning rate relative to the super saccharin reinforcers.

Overall, the analysis presented here supports the view that evaluation of within-session topographical data can provide further insight into the motivational profile of animals performing ratio schedules (Bradshaw & Killeen, 2012; Hutsell & Newland, 2013; Killeen, 1994; Ward et al., 2011). The present study also provides further validation of the rodent touchscreen apparatus as a tool for the implementation of ratio schedules (Heath et al., 2015, 2016) and demonstrates the utility of the expanded analytical approach. The original purpose of the PR schedule was to measure reward strength (Hodos, 1961) and in this study we have also confirmed that the touchscreen PR schedule is sensitive to differences in reward characteristics.

### Reinforcer choice in rodent touchscreen testing

To our knowledge this study reports for the first time a direct comparison between distinct liquid reinforcers in the touchscreen apparatus. The results indicate that using strawberry milkshake as a reinforcer can result in a more vigorous and sustained behavioral profile, consistent with previous studies indicating that milk-based liquid reinforcers have a higher reinforcer efficacy than sweetened pellet-based reinforcers (Hutsell & Newland, 2013). Additionally, these data indicate that mice are sensitive to the qualitative properties of liquid reinforcers under different ratio schedules in the touchscreen apparatus. This was dependent on whether an FR or PR schedule was used. The divergence in performance between these schedules indicates that the patterns of results obtained may yield different interpretations of performance and that both should be used in combination to assess the impact of experimental manipulations on behavior. Though we have not exhaustively examined the possible parameter space with respect to choice between liquid reinforcers, and previous studies have assessed choice between a greater number of food reinforcers (Biedermann, Garlick, & Blaisdell, 2012), our results are of practical use to researchers who wish to select an effective reinforcer for operant behavioral studies. Additionally, the results presented here provide a framework for future studies that seek to compare control of behavior and cognition under multiple reinforcers.

Overall, the results show that strawberry milkshake is a more effective reinforcer than super saccharin on operant tasks for motivation, work output, and learning. Finally, the present study provides support for the view that within-session analysis of response rate may be used to elucidate underlying patterns of behavior and can function as a highly useful adjunct to commonly used measures such as breakpoint and trials completed.
